# Supplementary material for: Effects of alcohol consumption on the prevalence and incidence of non-alcoholic fatty liver disease: A systematic review and meta-analysis
Source: PLoS One. 2025 Sep 19;20(9):e0330105. doi: 10.1371/journal.pone.0330105 (PMC12448959; doi:10.1371/journal.pone.0330105)
Supplement: S1 File — S1 Appendix. Complete list of search terms. S1 Table. The quality appraisal of prevalence studies. S2 Table. The quality appraisal of incidence studies. S1 Fig. Funnel plot analysis of publication bias for the incidence of NAFLD. S2 Fig. Funnel plot analysis of publication bias in male NAFLD prevalence. S3 Fig. Funnel plot analysis of publication bias in female NAFLD prevalence. S4 Fig. Funnel plot analysis of publication bias for the prevalence of NAFLD. (ZIP) [file pone.0330105.s001.zip › Supporting Information/S1 Appendix. Complete list of search term.docx]

**S1 Appendix. Complete list of search term**

****pubmed****

(("Non-alcoholic Fatty Liver Disease"[MeSH Terms] OR "Fatty Liver"[MeSH Terms] OR "NAFLD"[Title/Abstract] OR "NASH"[Title/Abstract] OR "liver fatty"[Title/Abstract] OR (("steato*"[Title/Abstract] OR "nonalcoholic fatty"[Title/Abstract]) AND ("hepat*"[Title/Abstract] OR "Liver"[Title/Abstract]))) AND ("Prevalence"[MeSH Terms] OR "Incidence"[MeSH Terms] OR "Incidence"[Title/Abstract] OR "Prevalence"[Title/Abstract]) AND ("Alcohols"[MeSH Terms] OR "Ethanol"[MeSH Terms] OR "Alcohols"[Title/Abstract] OR "Ethanol"[Title/Abstract] OR "Alcohol"[Title/Abstract])) AND (1970:2023[pdat])

**Embase**

Session Results

.......................................................

No. Query Results Results Date

#12. #3 AND #7 AND #11 5,680 2 Jan 2024

#11. #8 OR #9 OR #10 2,820,190 2 Jan 2024

#10. 'incidence':ab,kw,ti OR 'prevalence':ab,kw,ti 2,409,990 2 Jan 2024

#9. 'incidence'/exp OR 'incidence' 1,568,684 2 Jan 2024

#8. 'prevalence'/exp OR 'prevalence' 1,432,524 2 Jan 2024

#7. #4 OR #5 OR #6 151,405 2 Jan 2024

#6. 'non alcoholic fatty liver disease':ab,kw,ti OR 109,576 2 Jan 2024

'non-alcoholic fatty liver disease':ab,kw,ti OR

'steato*':ab,kw,ti OR 'nonalcoholic

fatty':ab,kw,ti OR 'liver, fatty':ab,kw,ti OR

'nash':ab,kw,ti OR 'nafld':ab,kw,ti

#5. 'fatty liver'/exp OR 'fatty liver' 128,706 2 Jan 2024

#4. 'nonalcoholic fatty liver'/exp OR 'nonalcoholic 75,431 2 Jan 2024

fatty liver'

#3. #1 OR #2 813,080 2 Jan 2024

#2. 'alcohol':ab,kw,ti OR 'ethanol':ab,kw,ti OR 605,968 2 Jan 2024

'alcohols':ab,kw,ti

#1. 'alcohol'/exp OR 'alcohol' 750,858 2 Jan 2024

****Web of science****

# Searches:

1: TS=(Non-alcoholic Fatty Liver Disease OR Fatty Liver OR NAFLD OR NASH) Results: 207999

2: TS=(Steato* OR Nonalcoholic Fatty) Results: 96384

3: TS=(hepat* OR liver) Results: 2351360

4: #2 AND #3 Results: 89098

5: TS=(Alcohols OR Ethanol OR Alcohol) Results: 1223916

6: TS=(Prevalence OR Incidence) Results: 2781817

7: #4 OR #1 Results: 223300

8: #7 AND #5 AND #6 Timespan: 1900-01-01 to 2023-12-31 Results: 3160

**Cochrane Library**

ID Search Hits

#1 MeSH descriptor: [Alcohols] explode all trees 42070

#2 MeSH descriptor: [Ethanol] explode all trees 5746

#3 MeSH descriptor: [Alcohol Drinking] explode all trees 5198

#4 (Alcohols or Ethanol or Alcohol):ti,ab,kw 34486

#5 MeSH descriptor: [Non-alcoholic Fatty Liver Disease] explode all trees 1669

#6 MeSH descriptor: [Fatty Liver] explode all trees 2184

#7 ((((hepat* or liver) and (Steato* or Nonalcoholic Fatty)) or Liver, Fatty or Nash or NAFLD)):ti,ab,kw 7795

#8 MeSH descriptor: [Prevalence] explode all trees 8724

#9 MeSH descriptor: [Incidence] explode all trees 15139

#10 (Incidence or Prevalence):ti,ab,kw 187460

#11 (#1 or #2 or #3 or #4) and (#5 or #6 or #7) and (#8 or #9 or #10) 122
