# Supplementary material for: Effects of alcohol consumption on the prevalence and incidence of non-alcoholic fatty liver disease: A systematic review and meta-analysis
Source: PLoS One. 2025 Sep 19;20(9):e0330105. doi: 10.1371/journal.pone.0330105 (PMC12448959; doi:10.1371/journal.pone.0330105)
Supplement: S1 File — S1 Appendix. Complete list of search terms. S1 Table. The quality appraisal of prevalence studies. S2 Table. The quality appraisal of incidence studies. S1 Fig. Funnel plot analysis of publication bias for the incidence of NAFLD. S2 Fig. Funnel plot analysis of publication bias in male NAFLD prevalence. S3 Fig. Funnel plot analysis of publication bias in female NAFLD prevalence. S4 Fig. Funnel plot analysis of publication bias for the prevalence of NAFLD. (ZIP) [file pone.0330105.s001.zip › Supporting Information/Table S1. The quality appraisal of prevelance studies.docx]

**Table S1. The quality appraisal of prevelance studies**

|  | Define the source of information (survey, record review) | List inclusion and exclusion criteria for exposed and unexposed subjects (cases and controls) or refer to previous publications | Indicate time period used for identifying patients | Indicate whether or not subjects were consectutive if not population-based | Indicate if evaluators of subjective components of study were masked to other aspects of the status of the participants | Describle any assessments undertaken for quality assurance purposes (e.g., test/retest of primary outcome measurements) | Explain any patient exclusions from analysis | Describle how confounding was assessed and/or controlled | If applicable, explain how missing data were handled in the analysis | Summarize patient response rates and completeness of data collection | Clarify what follow-up, if any, was expected and the percentage of patients for which incomplete data of follow-up was obtained | Total |
| --- | --- | --- | --- | --- | --- | --- | --- | --- | --- | --- | --- | --- |
| Caballerı´a (2010) | Y | Y | Y | NO | Y | Not reported | Y | Y | Y | Y | Y | 9 |
| 土居忠  (2010) | Y | Y | Y | Y | Not reported | Not reported | Y | Y | Y | Y | Y | 9 |
| Hamabe  (2011) | Y | Y | Y | Y | Y | Not reported | Y | Y | Y | Y | Y | 10 |
| Hamaguchi  (2005) | Y | Y | Y | NO | Y | Not reported | Y | Y | Y | Y | Y | 9 |
| Hara  (2019) | Y | Y | Y | Not reported | Y | Not reported | Y | Y | Y | Y | Y | 9 |
| Kächele  (2014) | Y | Y | NO | NO | Y | Not reported | Y | Y | Y | Y | Y | 8 |
| Lau (2015) | Y | Y | Y | Not reported | Y | Not reported | Y | Y | Y | Y | Y | 9 |
| Liu  (2014) | Y | Y | Y | NO | Y | Not reported | Y | Y | Y | Y | Y | 9 |
| Moriya  (2010) | Y | Y | Y | Not reported | Y | Y | Y | Y | Y | Y | Y | 10 |
| Moriya  (2013) | Y | Y | Y | NO | Y | Y | Y | Y | Y | Not reported | Y | 9 |
| Tan  (2020) | Y | Y | Y | Not reported | Y | NO | Y | Y | Y | Y | Y | 9 |
| Wong  (2012) | Y | Y | Y | NO | Y | Not reported | Y | Y | Y | Y | Y | 9 |
| Yamada  (2009) | Y | Y | Y | Y | Y | Not reported | Y | Y | Y | Y | Y | 10 |
| Cotrim  (2009) | Y | Y | Y | NO | Y | Not reported | Y | Y | Y | Y | Y | 9 |
