# Supplementary material for: Effects of alcohol consumption on the prevalence and incidence of non-alcoholic fatty liver disease: A systematic review and meta-analysis
Source: PLoS One. 2025 Sep 19;20(9):e0330105. doi: 10.1371/journal.pone.0330105 (PMC12448959; doi:10.1371/journal.pone.0330105)
Supplement: S1 File — S1 Appendix. Complete list of search terms. S1 Table. The quality appraisal of prevalence studies. S2 Table. The quality appraisal of incidence studies. S1 Fig. Funnel plot analysis of publication bias for the incidence of NAFLD. S2 Fig. Funnel plot analysis of publication bias in male NAFLD prevalence. S3 Fig. Funnel plot analysis of publication bias in female NAFLD prevalence. S4 Fig. Funnel plot analysis of publication bias for the prevalence of NAFLD. (ZIP) [file pone.0330105.s001.zip › Supporting Information/Table S2. The quality appraisal of incidence studies.docx]

**Table S2. The quality appraisal of incidence studies**

| study | Representativeness of the exposed cohort | Selection of the non exposed cohort | Ascertainment of exposure | Demonstration that outcome of interest was not present at start of study | Comparability of cohorts on the basis of the design or analysis | Assessment of outcome | Was follow-up long enough for outcomes to occur | Adequacy of follow up of cohorts | Quality  scope |
| --- | --- | --- | --- | --- | --- | --- | --- | --- | --- |
| Chang  (2020) | * | * | * | * | * | * | * | * | 8 |
| Hamabe  (2011) | * | * | * | * | * | * | * | * | 8 |
| Peeraphatdit  (2020) | * | * | * | * | * | * | * | * | 8 |
| Yamada  (2009) | * | * | * | * |  | * | * | * | 7 |
