# Supplementary figures and images for: Effects of alcohol consumption on the prevalence and incidence of non-alcoholic fatty liver disease: A systematic review and meta-analysis
Source: PLoS One. 2025 Sep 19;20(9):e0330105. doi: 10.1371/journal.pone.0330105 (PMC12448959; doi:10.1371/journal.pone.0330105)

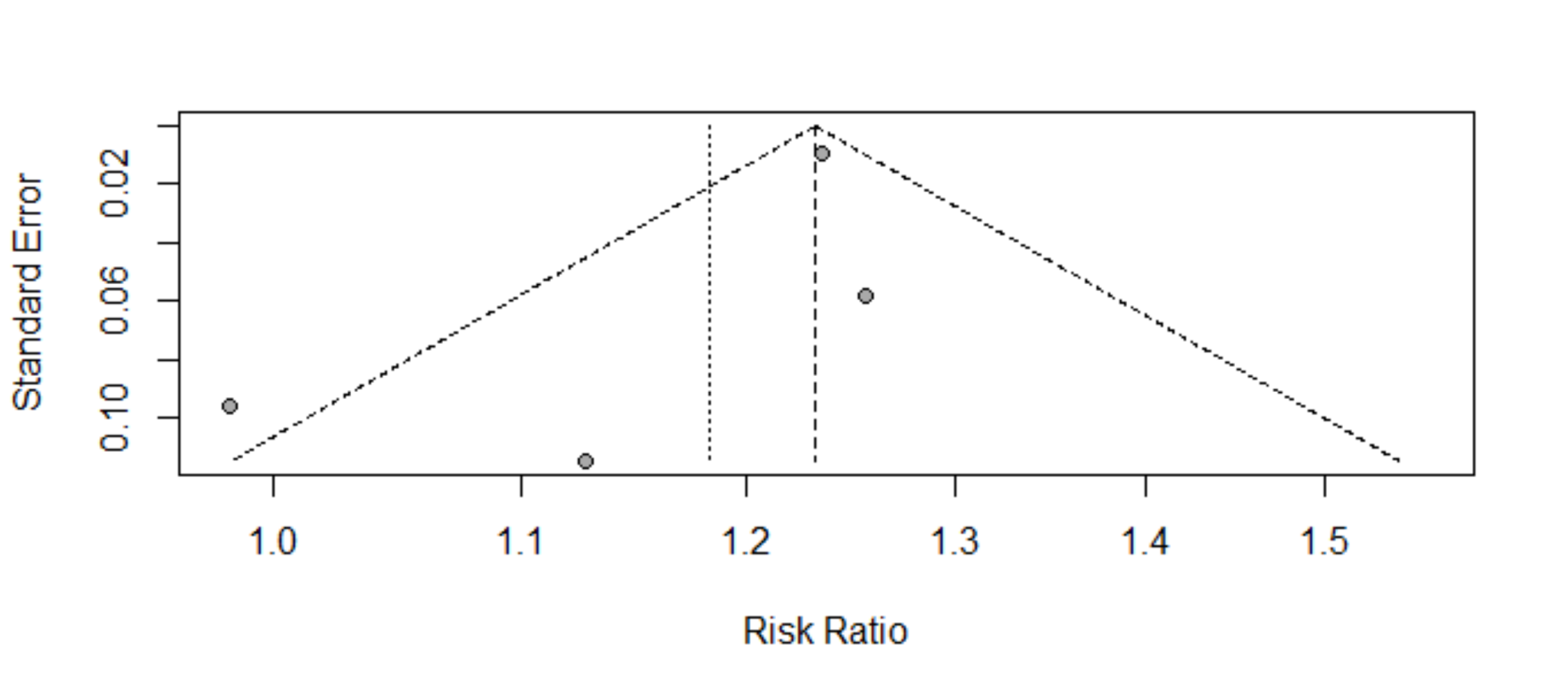

Supplement: S1 File — S1 Appendix. Complete list of search terms. S1 Table. The quality appraisal of prevalence studies. S2 Table. The quality appraisal of incidence studies. S1 Fig. Funnel plot analysis of publication bias for the incidence of NAFLD. S2 Fig. Funnel plot analysis of publication bias in male NAFLD prevalence. S3 Fig. Funnel plot analysis of publication bias in female NAFLD prevalence. S4 Fig. Funnel plot analysis of publication bias for the prevalence of NAFLD. (ZIP) [file pone.0330105.s001.zip › Supporting Information/Fig S1.Funnel plot analysis of publication bias for the incidence of NAFLD.tif]

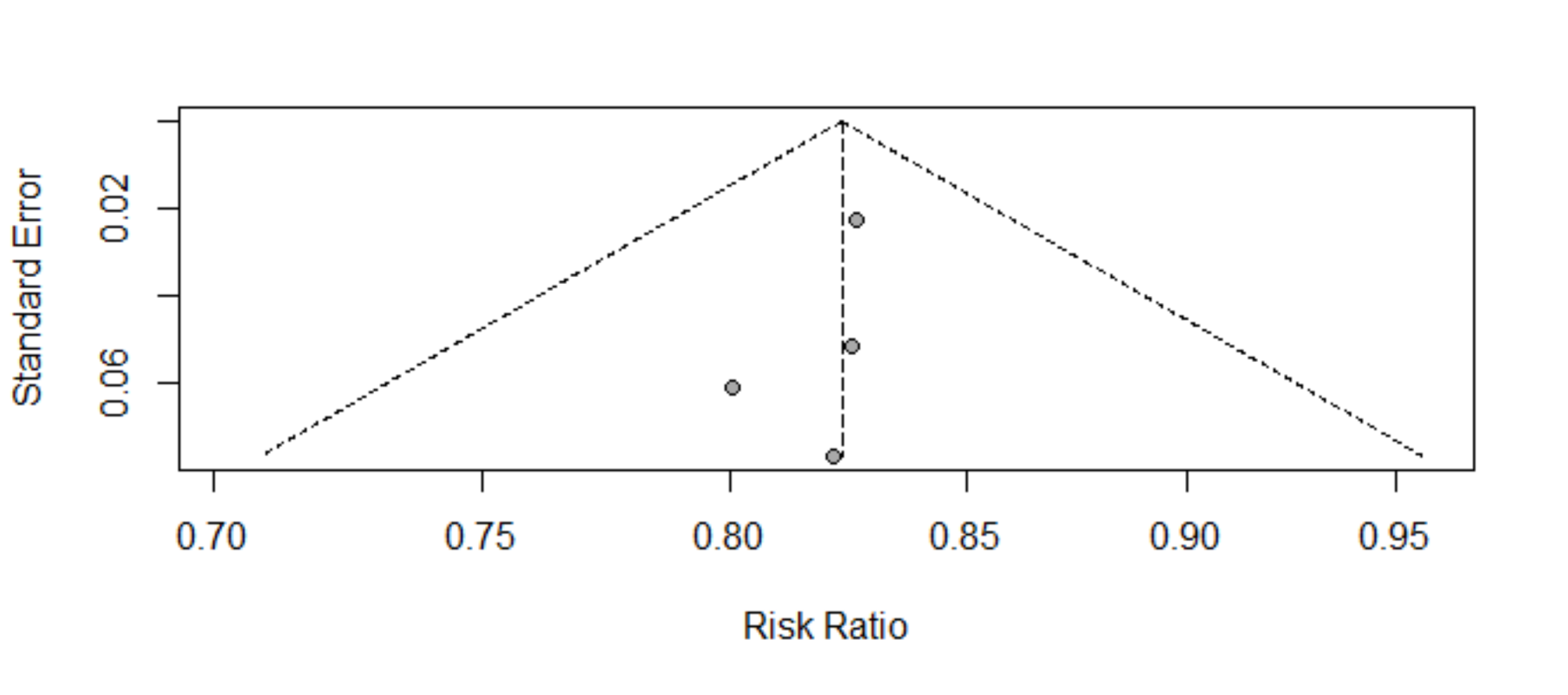

Supplement: S1 File — S1 Appendix. Complete list of search terms. S1 Table. The quality appraisal of prevalence studies. S2 Table. The quality appraisal of incidence studies. S1 Fig. Funnel plot analysis of publication bias for the incidence of NAFLD. S2 Fig. Funnel plot analysis of publication bias in male NAFLD prevalence. S3 Fig. Funnel plot analysis of publication bias in female NAFLD prevalence. S4 Fig. Funnel plot analysis of publication bias for the prevalence of NAFLD. (ZIP) [file pone.0330105.s001.zip › Supporting Information/Fig S2. Funnel plot analysis of publication bias in male NAFLD prevalence.tif]

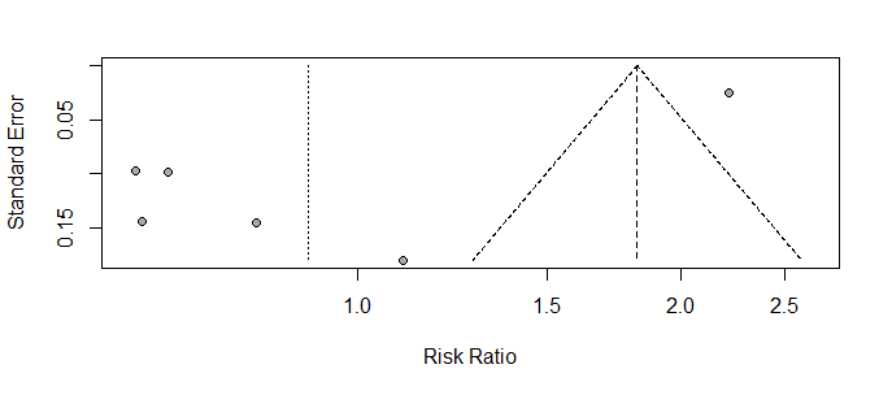

Supplement: S1 File — S1 Appendix. Complete list of search terms. S1 Table. The quality appraisal of prevalence studies. S2 Table. The quality appraisal of incidence studies. S1 Fig. Funnel plot analysis of publication bias for the incidence of NAFLD. S2 Fig. Funnel plot analysis of publication bias in male NAFLD prevalence. S3 Fig. Funnel plot analysis of publication bias in female NAFLD prevalence. S4 Fig. Funnel plot analysis of publication bias for the prevalence of NAFLD. (ZIP) [file pone.0330105.s001.zip › Supporting Information/Fig S3. Funnel plot analysis of publication bias in female NAFLD prevalence.tif]

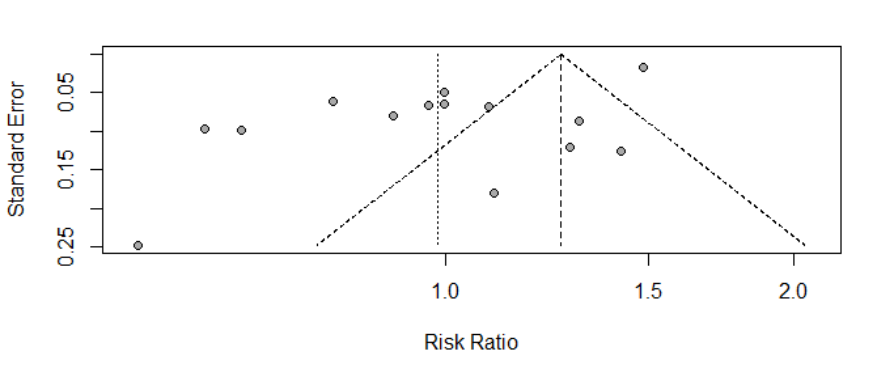

Supplement: S1 File — S1 Appendix. Complete list of search terms. S1 Table. The quality appraisal of prevalence studies. S2 Table. The quality appraisal of incidence studies. S1 Fig. Funnel plot analysis of publication bias for the incidence of NAFLD. S2 Fig. Funnel plot analysis of publication bias in male NAFLD prevalence. S3 Fig. Funnel plot analysis of publication bias in female NAFLD prevalence. S4 Fig. Funnel plot analysis of publication bias for the prevalence of NAFLD. (ZIP) [file pone.0330105.s001.zip › Supporting Information/Fig S4. Funnel plot analysis of publication bias for the prevelance of NAFLD.tif]
