# Supplementary material for: Effects of alcohol consumption on the prevalence and incidence of non-alcoholic fatty liver disease: A systematic review and meta-analysis
Source: PLoS One. 2025 Sep 19;20(9):e0330105. doi: 10.1371/journal.pone.0330105 (PMC12448959; doi:10.1371/journal.pone.0330105)
Supplement: Attachment — Submitted filename: A rebuttal letter2.docx [file pone.0330105.s002.docx]

Jingkai Yuan^1^, Zhiqi Yao^2^

^1^The Second Clinical College of Guangzhou University of Chinese Medicine, Guangzhou, China

^2^The Affiliated Shunde Hospital of Jinan University, Foshan, China

Email: [yaozhiqi@sddermyy.com](mailto:yaozhiqi@sddermyy.com) (ZQY)

Date: Feb.25, 2025

Dear Editors:

Thank you for your valuable feedback and for the opportunity to revise and resubmit our manuscript entitled "Effects of alcohol consumption on the prevalence and incidence of non-alcoholic fatty liver disease: a systematic review and meta-analysis". We greatly appreciate the time and effort you and the reviewers have taken to evaluate our work. We have carefully considered all of the comments and suggestions, and we believe that the revisions have significantly improved the quality and clarity of our manuscript.

Below, we provide a detailed response to each of the reviewers’ comments and outline the changes made:

1.) A numbered table of all studies identified in the literature search, including those that were excluded from the analyses.

a. For every excluded study, the table should list the reason(s) for exclusion.

We have revised the "Literature retrieved" section in accordance with the journal's requirements. However, as the specific reasons for exclusion were not systematically documented during the initial literature screening phase, we are unable to provide detailed justifications for the exclusion of each of the 5987 articles. Accordingly, we have classified the exclusion reasons as follows: 5985 articles were categorized as "not relevant" to the research topic, and the remaining 2 articles were classified as "withdrawal".

We hope that these revisions address the concerns raised and improve the manuscript. We are grateful for the constructive feedback and believe that it has strengthened our work. Please find the revised manuscript attached for your consideration.

Thank you again for your time and support. We look forward to your response.

Sincerely,

Jingkai Yuan^1^, Zhiheng Chen^2^, Yukai Gu^3^, Yaobin Liang^1^, Zhiqi Yao^4^

1.The Second Clinical College of Guangzhou University of Chinese Medicine, Guangzhou, China

2.The Affiliated TCM Hospital of Guangzhou Medical University, Guangzhou, China

3.The First Clinical College of Guangzhou University of Chinese Medicine, Guangzhou, China

4.The Affiliated Shunde Hospital of Jinan University, Foshan, ChinaCorresponding

Position: No.232 Waihuan East Road, University Town, Panyu District, Guangzhou, China

Author’s Email Address:[yaozhiqi@sddermyy.com](mailto:yaozhiqi@sddermyy.com) (ZQY)
