# Supplementary material for: Effects of alcohol consumption on the prevalence and incidence of non-alcoholic fatty liver disease: A systematic review and meta-analysis
Source: PLoS One. 2025 Sep 19;20(9):e0330105. doi: 10.1371/journal.pone.0330105 (PMC12448959; doi:10.1371/journal.pone.0330105)
Supplement: Attachment — Submitted filename: A rebuttal letter.docx [file pone.0330105.s003.docx]

Jingkai Yuan^1^, Zhiqi Yao^2^

^1^The Second Clinical College of Guangzhou University of Chinese Medicine, Guangzhou, China

^2^The Affiliated Shunde Hospital of Jinan University, Foshan, China

Email: [yaozhiqi@sddermyy.com](mailto:yaozhiqi@sddermyy.com) (ZQY)

Date: June.4, 2025

Dear Editors:

Thank you for your valuable feedback and for the opportunity to revise and resubmit our manuscript entitled "Effects of alcohol consumption on the prevalence and incidence of non-alcoholic fatty liver disease: a systematic review and meta-analysis". We greatly appreciate the time and effort you and the reviewers have taken to evaluate our work. We have carefully considered all of the comments and suggestions, and we believe that the revisions have significantly improved the quality and clarity of our manuscript.

Below, we provide a detailed response to each of the reviewers’ comments and outline the changes made:

1) Reviewer #1: Reviewer comment and suggestions

I was able to examine closely and realize that the author has been able to give paths for each step of writing the procedures in manuscript; however there are a few things authors need to include and make it clear. Use Level 1 heading for all major sections (Abstract, Introduction, Materials and methods, Results, Discussion, etc.).

• Adhere to the journals guideline in organizing the work especially PLOS ONE

ABSTRACT the authors should make the conclusions according to result.

RESULT the authors should arrange the section of the result well. Review and improve.

Answer: We have reorganized the Abstract, Introduction, Materials and Methods, Results, and Discussion into Level one heading . And we have amended the conclusions according to the result and arranged the section of the result.

2)Reviewer #2: Dear editor

Although many issues raised by reviewers are responsed; i think that as the included studies are analytical, it is important to add the resukt and adjustment variables for each study in table q; or oresent these data as a supplementary table.

Answer：Thank you for your question. Our study only collected the total number of participants and the number of cases of disease and/or incidence for each study. As such, the impact of the resukt and adjustment variables within each study on our research is relatively minor. Therefore, we believe that no additional information is necessary. However, if the journal feels that further supplementation would be beneficial, we are happy to make any necessary revisions.

We hope that these revisions address the concerns raised and improve the manuscript. We are grateful for the constructive feedback and believe that it has strengthened our work. Please find the revised manuscript attached for your consideration.

Thank you again for your time and support. We look forward to your response.

Sincerely,

Jingkai Yuan^1^, Zhiheng Chen^2^, Yukai Gu^3^, Yaobin Liang^1^, Zhiqi Yao^4^

1.The Second Clinical College of Guangzhou University of Chinese Medicine, Guangzhou, China

2.The Affiliated TCM Hospital of Guangzhou Medical University, Guangzhou, China

3.The First Clinical College of Guangzhou University of Chinese Medicine, Guangzhou, China

4.The Affiliated Shunde Hospital of Jinan University, Foshan, ChinaCorresponding

Position: No.232 Waihuan East Road, University Town, Panyu District, Guangzhou, China

Author’s Email Address:[yaozhiqi@sddermyy.com](mailto:yaozhiqi@sddermyy.com) (ZQY)
